# Dementia prevalence among hospitalized older patients: a multicenter study in Iran

**DOI:** 10.1590/1980-5764-DN-2023-0083

**Published:** 2024-03-11

**Authors:** Athena Enderami, Vahid Rashedi, Seyed Kazem Malakouti, Behnam Shariati, Najmeh Farhadi Birgani, Manouchehr Gharaeipour, Zeinab Kodan, Maryam Pourshams

**Affiliations:** 1 Mazandaran University of Medical Sciences, Faculty of Medicine, Department of Psychiatry, Sari, Mazandaran, Iran.; 2 University of Social Welfare and Rehabilitation Sciences, Iranian Research Center on Aging, Department of Aging, Tehran, Iran.; 3 Iran University of Medical Sciences, Director School of Behavioral Sciences and Mental Health, Tehran, Iran.; 4 Iran University of Medical Sciences, School of Medicine, Psychosocial Health Research Institute, Mental Health Research Center, Department of Psychiatry, Tehran, Iran.; 5 Golestan Hospital, Department of Psychiatry, Ahvaz, Iran.; 6 Hazrat Akram Rasoul Hospital, Department of Psychiatry, Tehran, Iran.; 7 Department of Psychiatry, Faculty of Nursing and Midwifery, Mazandaran University of Medical Sciences and Health Service, Sari, Mazandaran, Iran.; 8 Department of Psychiatry, Golestan Hospital, Ahvaz Jundishapur University of Medical Sciences, Ahvaz, Iran.

**Keywords:** Mental Status and Dementia Tests, Dementia, Aged, Prevalence, Testes de Estado Mental e Demência, Demência, Idoso, Prevalência

## Abstract

Dementia poses a significant societal and health challenge in the 21st century, with many hospitalized patients experiencing dementia without a documented diagnosis.

**Objective:**

To evaluate the prevalence of dementia and its associated risk factors among older patients admitted to hospitals.

**Methods:**

The study included older patients (≥ 60 years) admitted to medical departments of a general hospital in three major Iranian cities. Researchers utilized the Activities of Daily Living-Instrumental Activities of Daily Living (ADL-IADL) scale, the Geriatric Depression Scale (GDS), the Mini-Cog test, the 4 A’s test (4AT), and the Abbreviated Mental Test Score (AMTS). Among the 420 recruited older inpatients, 228 (54.3%) were female.

**Results:**

The mean age of participants was 71.39 years (standard deviation ±7.95), with 30.7% diagnosed with major neurocognitive disorder (dementia). The likelihood of dementia exhibited statistically significant correlations with gender, age, number of children, and occupation.

**Conclusions:**

Screening older individuals for cognitive impairment upon hospital admission holds the potential to prevent adverse outcomes and enhance the quality of treatment for patients concurrently dealing with dementia.

## RESUMO

A demência representa um grande desafio social e de saúde no século 21, com muitos pacientes hospitalizados sofrendo de demência sem um diagnóstico documentado.

**Objetivo:**

Avaliar a prevalência de demência e seus fatores de risco associados entre pacientes idosos hospitalizados.

**Métodos:**

O estudo incluiu pacientes idosos (≥ 60 anos) internados em um hospital geral em três grandes cidades iranianas. Os pesquisadores utilizaram a escala de Atividades da Vida Diária-Atividades Instrumentais da Vida Diária (*Activities of Daily Living-Instrumental Activities of Daily Living* – ADL-IADL), a Escala de Depressão Geriátrica, o teste Mini-Cog, o teste dos 4 As (4AT) e o Pontuação do Teste Mental Abreviado (*Abbreviated Mental Test Score* – AMTS). Dos 420 idosos selecionados, 228 (54,3%) eram do sexo feminino.

**Resultados:**

A média de idade dos participantes foi de 71,39 anos (desvio padrão ±7,95), sendo 30,7% diagnosticados com transtorno neurocognitivo maior (demência). A probabilidade de demência apresentou correlações estatisticamente significativas com sexo, idade, número de filhos e ocupação.

**Conclusões:**

A triagem de idosos para comprometimento cognitivo na admissão hospitalar tem o potencial de prevenir resultados adversos e melhorar a qualidade do tratamento para pacientes que lidam simultaneamente com demência.

## INTRODUCTION

Over the past decades, different types of dementia, including Alzheimer’s disease, have dramatically increased as the population ages. The global number of dementia cases increased to 43.8 million in 2016 and is expected to reach 152 million by 2050^1^. In common with many countries around the world, Iran’s aging population is growing. Older adults constituted 9.6% of the population in 2016, and it is expected that the proportion of Iranians aged 60 years and over will increase to 10.5% by 2025 and to 21.7% by 2050^2^. It is predicted that 8–10% of Iran’s older adults will suffer from Alzheimer’s disease in the next two to three decades^3^.

Dementia is a major and growing worldwide health challenge and imposes an enormous burden on affected individuals, their families, caregivers, communities, and societies^4,5^. It is one of the main causes of incapacity and dependence in older persons, accounting for 11.9% of the years of life with disability caused by noncommunicable diseases, which is increasing with the global rise in life expectancy^5^.

Most dementia people lack a formal diagnosis, especially in low- and middle-income countries. Without a formal diagnosis, older adults miss out on available treatment and comprehensive care that includes support and healthcare services, which can reduce the rate of cognitive decline^6^. The diagnosis of dementia may be more accurate during hospitalization, and its detection in general hospitals enables proper care^7^. Approximately half of the patients at general hospitals are over 65 years old, and between 20–40% of older patients have dementia comorbidities^8^. Hospitalization of people with dementia causes higher rates of mortality, delirium, falls, functional decline, spatial disorientation, malnutrition, dehydration, and depression. Hence, staff needs more knowledge and training about dementia and person-centered dementia care^9^.

To the best of our knowledge, only one hospital in Iran has undertaken a hospital-based investigation into the prevalence of dementia^10^. Therefore, the primary objective of this study was to employ a multicenter approach to ascertain the prevalence of dementia and explore its associated factors among older patients admitted to various hospitals.

## METHODS

### Study design and participants

In this cross-sectional study, the population included was older inpatients (≥ 60 years) with signed informed consent forms and the capacity to respond to questions at three general hospitals: Rasoul-e Akram in Tehran (the capital city), Imam Khomeini in Sari (the country’s north), and Golestan in Ahvaz (the country’s southwest) during January, February, and March 2022. Patients admitted to intensive or coronary care units were not considered. Additionally, individuals with acute neurological or metabolic problems, delirium, and those with psychiatric illnesses admitted to the psychiatric ward were excluded. Patients who were non-Farsi speakers and had undergone surgery within the previous eight hours were ineligible for the trial. The researchers recorded demographic data in a questionnaire that included gender, age, educational status, marital status, job, number of children, history of psychiatric and medical problems as well as substance use and medication.

This study was approved by Ahvaz Jundishapur University of Medical Sciences (ref. no: IR.AJUMS.REC.1399.756) and was performed in accordance with the Declaration of Helsinki.

### Cognitive assessment

### 4A’s test (4AT)

The 4AT is a simple and quick screening bedside tool for detecting delirium, originally released in 2011 on a particular website. It takes less than two minutes to complete and contains four items: alertness, attention, brief psychological examination, and a fluctuating course (or acute changes). The total score is 12 and a score of four or more indicates delirium. In a meta-analysis of 17 investigations, including the Iranian version, the 4AT demonstrated better diagnostic accuracy for delirium^11^. Hospitalized older patients with a score of four or more were not included in the study.

### Abbreviated Mental Test Score (AMTS)

The AMTS is a 10-point screening tool for assessing cognitive decline^12^. The AMTS was able to effectively distinguish the dementia group from the non-dementia group, and the correlation between the Mini-Mental State Examination results and dementia diagnoses, according to the Diagnostic and Statistical Manual of Mental Disorders (DSM), was significant (p<0.001). The ratings of six and seven demonstrated the ideal harmony between sensitivity (respectively, 99 and 94%) and specificity (respectively, 85 and 86%). For older Iranian individuals, the Persian form of the AMTS is a reliable cognitive testing tool^13^.

### Mini-Cog test

Mini-Cog is a brief and simple cognitive screening tool that takes an average of three minutes to complete. Mini-Cog was made by S. Borson et al. in 2001 and was originally designed for primary care settings. It comprises a three-word memory test and a second clock drawing test. A score of zero to two out of five is positive for cognitive impairment^14^. Rezaei et al. demonstrated the validity and reliability of Mini-Cog in older Iranian individuals^15^.

Patients who scored less than three out of five on the Mini-Cog test or less than eight out of 10 on the AMTS were evaluated by two geriatric psychiatrists to confirm the diagnosis of dementia based on the Diagnostic and Statistical Manual of Mental Disorders, Fifth Edition (DSM-5)^16^.

### Assessment of depression

The GDS-15, a 15-item short variant of the Geriatric Depression Scale, was applied to evaluate depression. This questionnaire is employed to test older persons for clinical depression. Yesavage et al. created the scale in 1982. Total scores of 12–15, 9–11, and 5–8 indicate the existence of severe, moderate, or mild depression, respectively, whilst a total score of 0–4 is regarded as normal. The test comprises 15 yes/no questions^17^. The Persian version of GDS-15 validated by Malakouti et al. showed that a score of eight and greater out of 15 is a positive screen for depression^18^.

### Assessment of functional status

The functional state of the individuals was assessed using the Persian translation of the ADL-IADL scale. This scale evaluates the patient’s capacity for activities like showering, clothing, grooming, transporting, getting around inside, maintaining continence, feeding, using the phone, daily errands such as grocery shopping and cooking, housekeeping, using transportation, responsibility for one’s medications, and managing money. Patients and their informed carers rated each of the aforementioned items on a three-point Likert scale. The overall score is between zero and 30, with higher scores indicating better functional status^19^. Spector et al. validated the composite ADL-IADL scale’s psychometric qualities in 1998^20^.

### Statistical analysis

The obtained data were recorded in Statistical Package for Social Sciences (SPSS Inc., Chicago, Ill., USA) version 22.0 for Windows. Quantitative data were reported as mean and standard deviation and qualitative data as frequency. Student t-test was used to analyze qualitative data in both gender groups and demographic variables. The chi-square test was used to analyze qualitative data in gender groups and demographic variables. A p-value of less than 0.050 was considered a statistically significant level.

## RESULTS

Of the 420 older inpatients recruited, 192 (45.7%) were male and 228 (54.3%) were female. The participants’ ages ranged from 60 to 99 years, with a mean age of 71.39 years (standard deviation [SD] ±7.95). Additional demographic information about the patients and how they varied between the three institutions under investigation can be found in Tables 1 and 2. As shown in Tables 1 and 2, there was no statistically significant difference in these variables among the three cities (p>0.050).

**Table 1:** Number and duration of hospitalizations, and number of children of patients admitted to three general hospitals.

Variables	Tehran		Ahvaz		Sari		Total	
	Mean	Min/Max	Mean	Min/Max	Mean	Min/Max	Mean	Min/Max
Age	68	61/88	71	60/98	67	60/99	72	60/99
Number of hospitalizations per patient in the last year	2*	1/46	2*	1/15	1*	1/5	2*	1/46
Duration of hospitalization	4*	1/60	6*	1/90	4*	1/21	4*	1/90
Number of children	4*	0/10	6*	0/20	6*	2/10	5*	0/20

Note: *Median.

**Table 2:** Demographic characteristics of patients admitted to three general hospitals by frequency and percentage.

Variable	Tehran	Ahvaz	Sari	Total
	n (%)	n (%)	n (%)	n (%)
Gender				
Male	79 (52.7)	64 (53.3)	49 (32.7)	192 (45.7)
Female	71 (47.3)	56 (46.7)	101 (67.3)	228 (54.3)
Job				
Retired	54 (36.0)	66 (55.0)	32 (21.3)	152 (36.2)
Housewife	67 (44.7)	30 (25.0)	88 (58.7)	185 (44.0)
Employed	29 (19.3)	24 (20.0)	30 (20.0)	83 (19.8)
Marital status				
Married	104 (69.3)	73 (60.8)	84 (56.0)	261 (62.1)
Single/divorced/widowed	46 (30.7)	47 (39.2)	66 (44.0)	159 (37.9)
Education				
Illiterate	45(30.0)	71 (59.2)	116 (77.3)	232 (55.2)
Basic	57 (38.0)	18 (15.0)	8 (5.3)	83 (19.8)
School dropout	18 (12.0)	13 (10.8)	8 (5.3)	39 (9.3)
Diploma and university degree	30 (20.0)	18 (15.0)	18 (12.0)	66 (15.7)
Substance abuse				
No	116 (77.3)	93 (77.5)	107 (71.3)	316 (75.2)
Yes	20 (13.3)	4 (3.3)	3 (2.0)	27 (6.4)
Ward				
Internal	87 (58.0)	84 (70.0)	76 (50.7)	247 (58.8)
Surgery	63 (42.0)	36 (30.0)	74 (49.3)	173 (41.2)

According to the Mini-Cog test, 91 (21.7%) patients, and according to the AMTS instrument, 171 (40.7%) individuals had some level of cognitive impairment in their performance. A clinical interview based on the DSM-5 was applied to examine this group. Out of all patients, 129 were diagnosed with dementia (a major neurocognitive disorder) and the others did not meet the criteria. The dementia prevalence was 30.7% in our investigation because cognitive abnormalities in the latter group were thought to result from mild cognitive impairment (MCI) or depression.

A statistically significant difference was found in the Mini-Cog, AMTS, and ADL-IADL scores between the demented and non-demented groups (p<0.001). The mean GDS-15 scores showed no significant differences between the demented and non-demented groups (Table 3).

**Table 3:** Test scores in patients with dementia compared to non-demented patients.

Variables	Mean±SD	p-value
IADL		
Demented	2.83±2.67	<0.001
Non-demented	10.42±3.98	
ADL		
Demented	8.20±3.96	<0.001
Non-demented	14.39±3.02	
AMTS		
Demented	4.81±0.85	<0.001
Non-demented	9.19±1.13	
GDS-15		
Demented	5.11±3.97	0.060
Non-demented	4.40±3.35	
Mini-Cog		
Demented	1.50±1.23	<0.001
Non-demented	3.58±1.46	

Abbreviations: SD, standard deviation; IADL, Instrumental Activities of Daily Living; ADL, Activities of Daily Living; AMTS, Abbreviated Mental Test Score; GDS-15, Geriatric Depression Scale 15-item; Mini-Cog, a brief and simple cognitive screening tool.

The findings also revealed a statistically substantial connection between gender, age, job, marital status, the number of children, educational status, and dementia prevalence (Table 4).

**Table 4:** Demographic characteristics in demented and non-demented patients (according to the Diagnostic and Statistical Manual of Mental Disorders, Fifth Edition).

Variables	n (%)		p-value	OR (95%CI)
	Demented	Non-demented		
Age (years)				
<71	32 (14.5)	189 (85.5)	<0.001	1.28 (1.17–1.40)
≥71	97 (48.7)	102 (51.3)		
Gender				
Male	27 (14.1)	165 (85.9)	<0.001	1.55 (1.36–1.77)
Female	102 (44.7)	126 (55.3)		
Marital status				
Married	42 (16.1)	219 (83.9)	<0.001	1.26 (1.14–1.39)
Single/divorced/widowed	87 (54.7)	72 (45.3)		
Education				
Undergraduate	128 (36.2)	226 (63.8)	<0.001	0.84 (0.79– 0.89)
Diploma/university degree	1 (1.5)	65 (98.5)		
Substance abuse				
Non-abuser	128 (32.6)	265 (67.4)	0.021	0.84 (0.81–0.88)
Abuser	1 (3.7)	26 (96.3)		
Employment				
Non-employed	121 (35.9)	216 (64.1)	<0.001	0.85 (0.80–0.91)
Employed	8 (9.6)	75 (90.4)		
Number of children				
<4	14 (14.1)	85 (85.9)	<0.001	1.33 (1.19–1.49)
≥4	114 (35.6)	206 (64.4)		

Abbreviations: OR, odds ratio; CI, confidence interval.

Dementia was 1.55 times more common in women than in men. Individuals 71 years old and older had a 1.28 times higher risk of dementia than patients under this age. A history of unemployment was also significantly linked to the disease. Furthermore, married people had a 1.26 times higher risk of developing dementia than unmarried, divorced, or widowed adults.

## DISCUSSION

This is the first multicenter study in Iran and the second to examine dementia among older patients transferred to general wards. According to the study’s findings, dementia affects 30.7% of patients treated in hospital non-psychiatric wards in the three largest cities of Sari, Ahvaz, and Tehran. The prevalence of the disease was not significantly different in the three cities assessed. It is noteworthy that none of these patients were diagnosed with dementia before this study.

The frequency of dementia in general hospital wards in developing nations has been investigated quantitatively. According to Kamalzadeh et al., the disease was found to be 22% prevalent in Rasoul-e Akram Hospital in Tehran^10^. In five general hospitals in Brazil, Maia et al. found a 17% prevalence of dementia^21^. Contrarily, according to the study by Dieu et al., it was substantially lower (8.8%) in Benin^22^. Nonetheless, it should be noted that this discrepancy could potentially be attributed to variations in the sampling methodology employed in that particular study, as it included not only older patients but also younger individuals referred to the neurology department for counseling purposes.

Compared to investigations in developed countries, the results of the present study are consistent with a recent study that identified a 21% prevalence of 493 older patients in the medical, surgical, and orthopedic wards at Queensland Teaching Hospital^23^. Similarly, Timmons et al. found that 25% of older patients receiving treatment in acute hospital wards in Ireland had dementia^24^. However, Briggs et al. realized that 38% of older patients referred to emergency units in Ireland had dementia^25^. Conversely, Kofahi et al. reported an annual result of 1.29% for dementia in people over 50 years of age receiving hospitalization in Jordan^26^. The low rate observed can be attributed to the relatively youthful demographic of the Jordanian population and the comparatively lower mean age of the hospitalized patients.

Another significant finding in the current study and other similar studies is that the incidence of dementia in general hospitals is significantly higher than the 6% recorded in the general population^27^. In 2014, Rashedi et al. reported that 2.8% of adults in a community had cognitive impairment^28^. Furthermore, a nationwide survey of older adults in Iran revealed a 7.9% overall prevalence of dementia in those over 60 years of age (8.7% in women and 6.5% in men)^29^. Some studies have shown that patients with cognitive impairment are among the populations that utilize general hospital services the most and that patients with dementia are associated with increased hospitalization rates because of any diagnosis in general hospital wards^30-32^. Multiple factors contribute to this observation. Primary conditions that elevate the risk of dementia, such as cerebrovascular disease, along with conditions that can be secondary to dementia, like urinary incontinence and dysphagia, may amplify the susceptibility to hospitalization. These secondary conditions, in turn, heighten the risk of complications such as urinary tract infections and pneumonia.

In addition, dementia is associated with the inability of individuals to manage their medical conditions and inform others about their condition and disease, which increases the risk of complications and the need for hospitalization^33^. The care challenges for this group of patients are significantly higher than for the group without cognitive impairment (87 vs. 24%). This may directly impact the quality and quantity of medical care these individuals receive in hospitals. Since many patients do not have a prior diagnosis of dementia, recognizing these challenges and educating the treatment staff and the patients’ families can prevent or reduce adverse consequences^34^.

The current study found that the prevalence of dementia increased with age, rising from 14.5% in patients under 71 years old to 48.7% in those aged 71 years and over. These findings are consistent with other investigations that reveal a connection between aging and the disease^10,28,35^. Additionally, dementia in females was 45%, significantly higher than in males (14%). These findings are consistent with other studies in this field, which show a higher prevalence in women. According to Calatayud et al., general cognitive function was reported to be significantly higher in men than in women^36^. Conversely, in a study by Klich-Rączka et al. in Poland, there was no difference when comparing gender, although the diagnostic suspicion of dementia was higher in men^37^. The difference between genders may stem from the interplay of genes, hormones, and socio-environmental factors.

The present study also found that patients with four or more children had a significantly higher prevalence of dementia than patients with fewer children. This observation is consistent with the findings of Kamalzadeh et al.^10^. Additionally, according to research by Beeri et al., women who have at least one child are more likely to develop Alzheimer’s disease than men who do not have children^38^. Colucci et al. reported similar findings^39^. This relationship, which has been shown in several studies, may be due to social and biological factors related to the role of women in raising children and family matters. It is well-established that a lack of estrogen increases the likelihood of developing Alzheimer’s disease^40^. Research has shown that the number of children and circulating estrogen levels are inversely correlated, with women who have more children having lower levels of circulating estrogen than those who have fewer or no children^41^. The depletion of estrogen resulting from multiple childbirths may be associated with the neuropathology of Alzheimer’s disease.

Another finding from the current study is that dementia rates were significantly lower in people who work outside the home (10%) compared to housewives and non-employed individuals (35%). This discrepancy may be attributed to differences in their premorbid functions and abilities rather than a protective effect of being employed against dementia. Some studies have demonstrated that not having an active job during a person’s lifetime increases the risk of cognitive impairment and dementia due to reduced cognitive reserves^42^. In the present investigations, the prevalence of dementia in married patients was significantly lower than in single/widowed/divorced patients (16 vs. 54%). This finding contrasts with the study conducted by Kamalzadeh et al., who encountered no difference in rates between married and unmarried patients^10^. However, most experts have stressed that the absence of social and family support is a risk factor for developing dementia^43^. Additionally, in the present work, the level of education was significantly associated with dementia; in patients with a diploma or higher, the prevalence of the disease was only 1.5%. This finding is in line with the results of most studies that show higher education is associated with a lower risk of cognitive impairment^21,44,45^.

The results of the current study, in conjunction with similar investigations conducted globally, reveal that a notable proportion of elderly patients admitted to general hospitals lack a pre-existing dementia diagnosis thereby elevating the risk of adverse outcomes. Additionally, it is observed that the caregiving challenges for this particular patient group are considerably more pronounced than for other cohorts. Although regular dementia screening is not recommended for all older people because of a lack of evidence, identifying cases of dementia in general hospitals can be a logical approach due to the many older patients with dementia who are admitted for other medical problems before a dementia diagnosis is made. Identifying cognitive impairment in hospitalized older patients and making a timely diagnosis, as well as educating the treatment staff and patients’ families about their care needs, can lead to proper care and reduce adverse consequences. Therefore, it is essential to create proper guidelines and educate different specialists on how to screen for and recognize dementia manifestations in hospitalized older patients.

In the present study, we observed no significant correlation between past medical history and the prevalence of dementia. This lack of association may be attributed to the reliance on self-reported medical history by the participants. It has been documented that a considerable number of individuals with underlying conditions such as diabetes and hypertension were unaware of their medical status in the context of Iran^46,47^.

The study demonstrates notable strengths, such as a well-defined sample size and clinical assessments performed by psychiatrists, ensuring a high level of diagnostic accuracy. Nonetheless, the study design presents a limitation. Prospective studies have the potential to provide more nuanced insights into the variables associated with dementia prevalence. To validate our results, further exploration through additional multicenter trials may be necessary.
